# Experimental Treatment with Edaravone in a Mouse Model of Spinocerebellar Ataxia 1

**DOI:** 10.3390/ijms241310689

**Published:** 2023-06-26

**Authors:** Martina Sucha, Simona Benediktova, Filip Tichanek, Jan Jedlicka, Stepan Kapl, Dana Jelinkova, Zdenka Purkartova, Jan Tuma, Jitka Kuncova, Jan Cendelin

**Affiliations:** 1 Department of Pathological Physiology, Faculty of Medicine in Pilsen, Charles University, alej Svobody 76, 323 00 Pilsen, Czech Republic; 2 Laboratory of Neurodegenerative Disorders, Biomedical Center, Faculty of Medicine in Pilsen, Charles University, alej Svobody 76, 323 00 Pilsen, Czech Republic; 3 Department of Physiology, Faculty of Medicine in Pilsen, Charles University, alej Svobody 76, 323 00 Pilsen, Czech Republic; 4 Mitochondrial Laboratory, Biomedical Center, Faculty of Medicine in Pilsen, Charles University, alej Svobody 76, 323 00 Pilsen, Czech Republic; 5 Laboratory of Experimental Neurophysiology, Biomedical Center, Faculty of Medicine in Pilsen, Charles University, alej Svobody 76, 323 00 Pilsen, Czech Republic

**Keywords:** cerebellum, edaravone, mitochondria, neurodegeneration, spinocerebellar ataxia type 1

## Abstract

Edaravone is a mitochondrially targeted drug with a suggested capability to modify the course of diverse neurological diseases. Nevertheless, edaravone has not been tested yet in the context of spinocerebellar ataxia 1 (SCA1), an incurable neurodegenerative disease characterized mainly by cerebellar disorder, with a strong contribution of inflammation and mitochondrial dysfunction. This study aimed to address this gap, exploring the potential of edaravone to slow down SCA1 progression in a mouse knock-in SCA1 model. SCA1^154Q/2Q^ and healthy SCA1^2Q/2Q^ mice were administered either edaravone or saline daily for more than 13 weeks. The functional impairments were assessed via a wide spectrum of behavioral assays reflecting motor and cognitive deficits and behavioral abnormalities. Moreover, we used high-resolution respirometry to explore mitochondrial function, and immunohistochemical and biochemical tools to assess the magnitude of neurodegeneration, inflammation, and neuroplasticity. Data were analyzed using (hierarchical) Bayesian regression models, combined with the methods of multivariate statistics. Our analysis pointed out various previously documented neurological and behavioral deficits of SCA1 mice. However, we did not detect any plausible therapeutic effect of edaravone on either behavioral dysfunctions or other disease hallmarks in SCA1 mice. Thus, our results did not provide support for the therapeutic potential of edaravone in SCA1.

## 1. Introduction

Spinocerebellar ataxia type 1 (SCA1) is a lethal neurodegenerative disease with autosomal dominant inheritance. To date, there is no effective therapy that would significantly delay the neurodegenerative process and the development of disability in SCA1 patients. SCA1 is caused by CAG repeat expansion (more than 40 CAG repeats) in the gene encoding ataxin-1 (*ATXN1*) protein [1], playing a role in the regulation of mitochondrial bioenergetics in the cerebellum [2]. The mutation leads to the accumulation of a protein with the expanded polyglutamine tract and its misfolding, aggregation, and formation of intranuclear inclusions that impair the function and viability of neurons [1,3]. *ATXN1* is widely expressed throughout the brain [1]. Therefore, the subsequent pathogenetic chain leads to dysfunction and degeneration of the cerebellum, brainstem, and hippocampus [4,5,6,7].

Complex neuropathology includes slowly progressive degeneration of cerebellar Purkinje cells [6], as well as a reduction in dendritic arborization of hippocampal neurons [4,8]. Suppression of neurogenesis and neuroplasticity [4,9], changes in the brain vascular system [10], and glial activation [11,12] participate in the neurodegenerative process. Furthermore, disruption of mitochondrial functions and increased production of reactive oxygen species (ROS) in the cerebellum have been found and might be not only a consequence of cell metabolism disorder but also a factor contributing to primary and/or secondary regressive changes and cell death [2,13,14]. 

As a consequence of cerebellar degeneration, both SCA1 mice and SCA1 patients develop cerebellar ataxia [15]. In patients with SCA1, oculomotor disorders, bulbar syndrome, and respiratory disorders have also been reported [16]. Nevertheless, SCA1 also leads to neuropsychiatric problems, including cognitive impairment, anxiety, apathy, and depression, which can be seen in both human and mouse models [17,18,19,20,21,22,23,24]. In addition, changes in functions associated with hippocampal plasticity have been described [9,25,26]. Hippocampal plasticity depends crucially on the proper functioning of mitochondria [27,28,29]. Tichanek et al. [4] pointed to significantly impaired mitochondrial function, specifically in the hippocampus of SCA1 mice, and suggested that mitochondrial dysfunction may significantly contribute to the hippocampal neuropathology in SCA1. Therefore, drugs targeting mitochondrial dysfunction and its consequences (e.g., increased oxidative stress) could potentially have a therapeutic effect in SCA1.

One of the useful approaches in searching for therapeutic possibilities is drug repurposing, which reduces timelines, risks, and costs compared to novel drug development [30]. Neuroprotective substances having a therapeutic effect in other neurological diseases have often been shown to help in some, but not all cerebellar diseases [31,32]. Studies in preclinical models can identify promising substances to be tested in human patients during clinical trials [33]. The pharmacodynamics, side effects, safety, etc., of such drugs are already known. Thus, there is a chance of a faster validation process before routine clinical use to treat cerebellar diseases. One of the potential drugs for this purpose could be edaravone (MCI-186).

In clinical practice, edaravone is used in acute ischemic stroke, since it reduces neurological deficits in patients [34,35,36]. Later it was approved for the treatment of amyotrophic lateral sclerosis (ALS) [37]. Surprisingly, edaravone has not yet been studied in connection with spinocerebellar ataxias. There are reasons that make edaravone a potential agent for improving the condition of patients with SCAs:

(1) Edaravone has been shown to be effective in other neurological diseases, namely stroke and ALS [35,36,37]; thus, it is a good candidate for drug repurposing studies. ALS in particular is a disease whose pathophysiology partially overlaps with SCA1 [38,39].
(2) Edaravone is a mitochondrial-targeting drug that acts as a free radical scavenger [40]. It protects neurons and various types of glia from oxidative stress-induced cell death [41,42,43,44].
(3) Mitochondrial function is disturbed in SCA1 [4,45], and mitochondria-targeted antioxidant MitoQ ameliorated mitochondrial activity, reduced oxidative stress-induced DNA damage and Purkinje cell loss, and improved motor coordination in SCA1 mice [45].
(4) Edaravone suppresses the inflammatory response [46], which also accompanies SCA1 [11].

**Figure 1:**
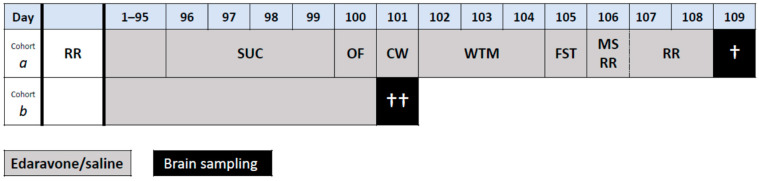
Scheme of the experiment for cohorts *a* and *b*. Day 1 represents the first day of treatment with edaravone or saline at the age of 8 weeks. Functional tests included the sucrose preference test (SUC), the open field test (OF), the gait analysis by CatWalk system (CW), the water T-maze (WTM), the forced swimming test (FST), the rotarod (RR), and the muscle strength measurement (MS). † Brain sampling for histological examination, †† Brain sampling for mitochondrial function examination and IL6 and BDNF level measurement.

Therefore, the study aimed to assess the therapeutic potential of chronic edaravone administration in heterozygous SCA1 mice with 154 CAG repeats (SCA1^154Q/2Q^), and healthy wild-type (WT) mice (SCA1^2Q/2Q^). For this purpose, we established four experimental groups of mice: SCA1^154Q/2Q^ edaravone-treated (SCA1_E), SCA1^154Q/2Q^ saline-treated (SCA1_0), SCA1^2Q/2Q^ edaravone-treated (WT_E), and SCA1^2Q/2Q^ saline-treated (WT_0). Each group consisted of two cohorts. One cohort was treated with edaravone or saline for 95 days, after which the mice were subjected to a 2-week series of experiments during which the treatment was still continued. After that, the mice were euthanized for the collection of brains for histological examination. The second cohort of mice was treated for 100 days. Then, the mice were euthanized for the collection of brains for mitochondrial function examination and biochemical analyses (Figure 1).

## 2. Results

Between-group differences are described using the probability of direction and, if this parameter is higher than 0.9, with odds ratio, absolute difference, or fold difference depending on the data type. Details for all reported differences, including those with a probability of direction less than 0.9, are presented in the figures.

### 2.1. Sucrose Preference Test 

**Figure 2:**
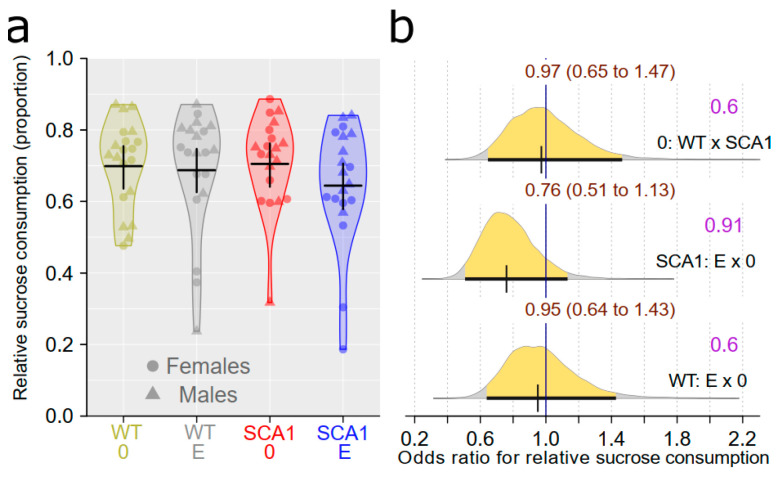
Sucrose preference test of anhedonia-like behavior. (**a**) The relative sucrose consumption in individual experimental groups (WT = wild-type mice, SCA1 = SCA1 mice, 0 = saline, E = edaravone), where lines represent group-specific prediction from the Bayesian model and its 95% credible intervals. (**b**) The posterior probability distribution for odds ratios (ORs) between relative sucrose consumption values in compared groups. Curves represent the posterior probability distribution, and lines below the curve show the 95% credible interval for the OR. The exact values for the estimated OR and its credible interval are shown in brown color (top), and the probability of direction is shown in purple color (right).

To assess the level of depressive-like traits and anhedonia, a sucrose preference test was performed. The effect of genotype in this test was uncertain, with the probability of direction only being 0.6 and with an odds ratio (WT vs. SCA1) of 0.97 (95% credible interval (CI): 0.65 to 1.47), indicating that the odds of relative sucrose consumption in WT mice were (with 95% probability) 0.65- to 1.47-fold larger in WT compared to SCA1 mice. Similarly, the edaravone effect was uncertain in the case of both genotypes. The probability of direction was 0.91 (odds ratio of 0.76) in SCA1 and only 0.6 in WT mice (Figure 2).

### 2.2. Open Field Test 

**Figure 3:**
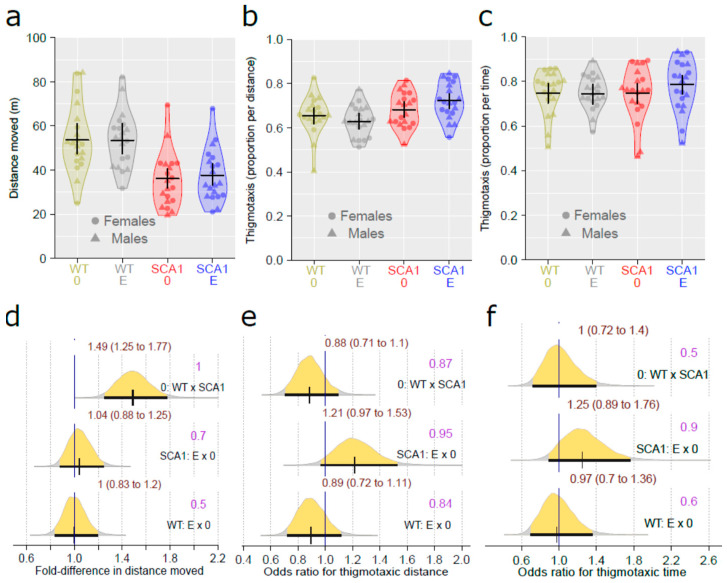
Open field test. (**a**) Spontaneous activity (distance moved), (**b**) thigmotaxis expressed as the portion of distance moved in the peripheral 6 cm wide zone (thigmotaxic distance), and (**c**) thigmotaxis expressed as the portion of time spent in the peripheral 6 cm wide zone (thigmotaxic time) in individual experimental groups (WT = wild-type mice, SCA1 = SCA1 mice, 0 = saline, E = edaravone), where lines represent group-specific prediction from the Bayesian model and its 95% credible intervals. (**d**) The posterior probability distribution for fold differences (FDs) between compared groups in distance moved. (**e**,**f**) The posterior probability distribution for odds ratio (OR) between compared groups in thigmotaxic distances and thigmotaxic time, respectively. Curves represent the posterior probability distribution, and lines below the curve show the 95% credible interval for the effect size (FD or OR). The exact values for the estimated effect size and its credible interval are shown in brown color (top), and the probability of direction is shown in purple color (right).

Spontaneous locomotor activity and preference for the periphery of an arena as a sign of anxiety were examined using the open field test. Wild-type mice walked 1.49 times (95% CI: 1.25 to 1.77, probability of direction: 1) longer distance compared to SCA1 mice. However, the effect of edaravone on walked distance was uncertain and probably negligible or small (probability of direction of 0.7 in SCA1 mice and 0.5 in WT mice; Figure 3a,d). Genotype (probability of direction for thigmotaxic distance was 0.87 and for thigmotaxic time 0.5) and treatment (for thigmotaxic distance, probability of direction was 0.95 with fold difference of 1.21 in SCA1 and 0.84 in WT mice; for thigmotaxic time, probability of direction was 0.9 with fold difference of 1.25 in SCA1 and 0.6 in WT mice) effects on thigmotaxis were both rather uncertain (Figure 3b,c,e,f). 

### 2.3. Gait Analysis

Spontaneous gait was examined and analyzed using the CatWalk system providing a number of gait parameters. The principal component analysis of gait parameters showed that 68% of the variance in the gait data might be expressed using three main PCs. The correlation between the three PCs and gait parameters is shown in Supplementary Material S1. PC1 correlates mainly with fast walking, high swing speed, and long stride. PC2 correlates with gait inconsistency (large standard deviations of the gait parameters). PC3 correlates mainly with support of only zero legs and one leg, and negatively with support of three legs. 

**Figure 4:**
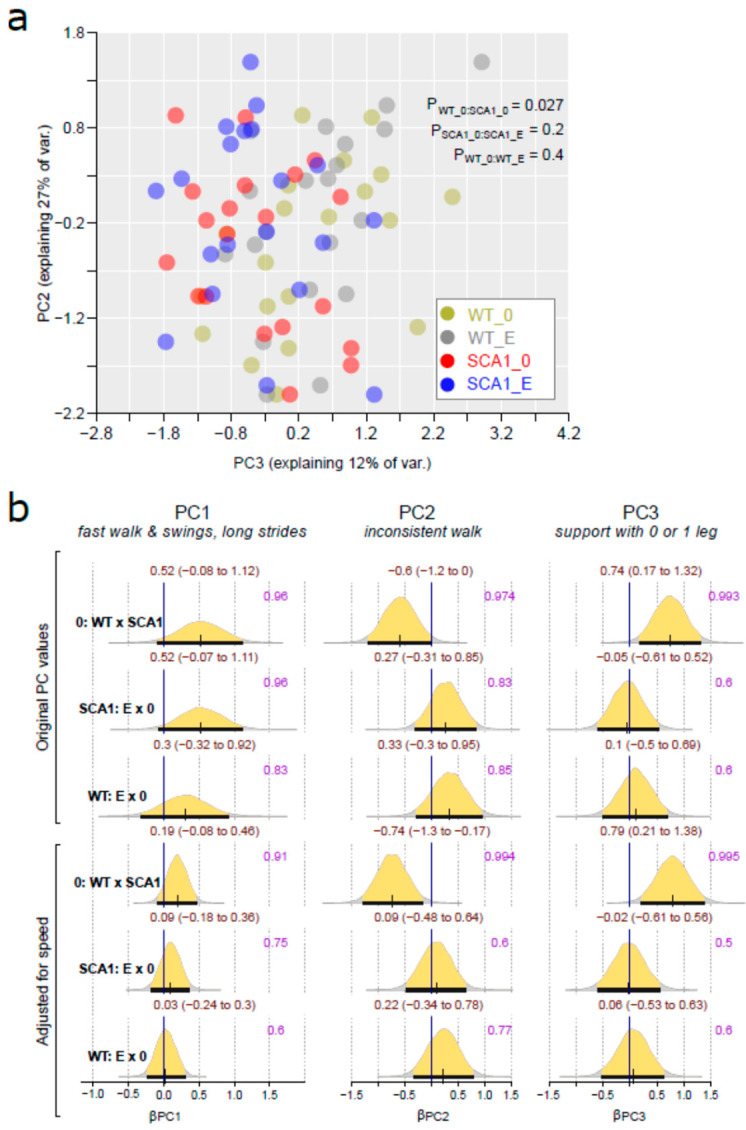
Gait analysis (CatWalk). (**a**) Relation between principal component 2 (PC2) and principal component 3 (PC3) values in individual mice from all experimental groups (WT = wild-type mice, SCA1 = SCA1 mice, 0 = saline, E = edaravone). (**b**) The posterior probability distribution for absolute difference in PC1, PC2, and PC3 in compared groups without (original PC values) or with adjusting for walking speed. Curves represent the posterior probability distribution, and lines below the curve show the 95% credible interval for the absolute difference. The exact values for the estimated difference and its credible interval are shown in brown color (top), and the probability of direction is shown in purple color (right).

The results of Bayesian multivariate regression suggested that PC1 values (Z-standardized) may be smaller in SCA1 mice (implying slower walking), with the posterior probability of the effect of 96%, implying an uncertain effect (95% CI for WT standardized effect: −0.08 to 1.12). The effect is even less certain when the walking speed was adjusted (Figure 4). PC2 was estimated to be lower by 0 to 1.2 (0.17 to 1.3 in speed-adjusted analysis) in WT mice, compared to SCA1, suggesting walk inconsistency in SCA1 mice. PC3 axes were higher in WT mice (95% CI for standardized effect: 0.17 to 1.32 in unadjusted and 0.21 to 1.38 in adjusted analysis), suggesting that SCA1 mice require the support of more legs for stable walking. The directions of the effects of edaravone on the PC1-PC3 were uncertain (Figure 4). 

The genotype-related differences in the gait were further supported with non-Bayesian PERMANOVA analysis (P for the difference between control WT and SCA1 mice = 0.027; Figure 4a). See Supplementary Material S2 for individual gait parameters, their median values across groups, and results of the Kruskal–Wallis test of group differences.

### 2.4. Water T-Maze

**Figure 5:**
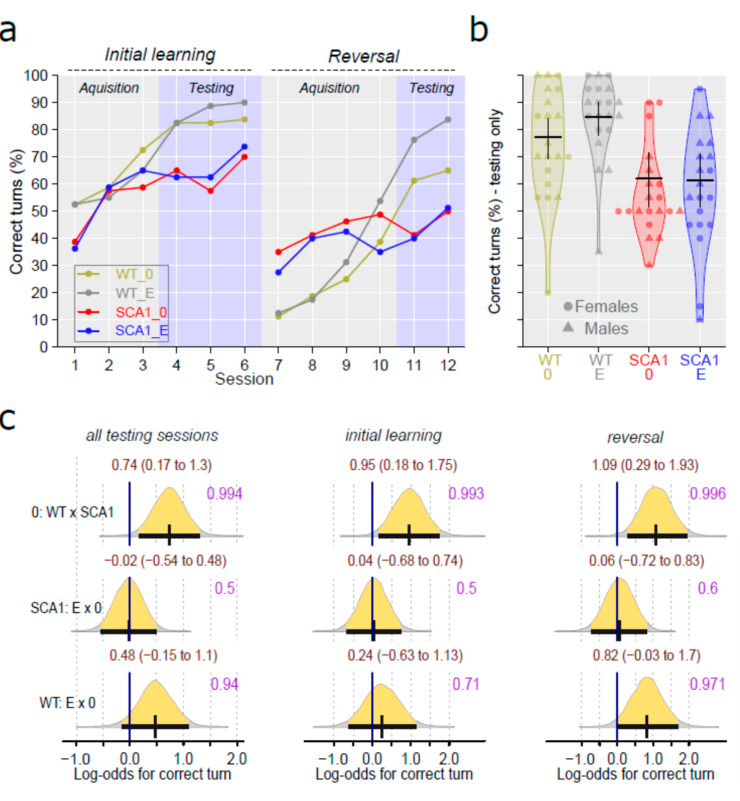
T-maze test. (**a**) Mean percentage of correct maze arm choices in individual initial learning and reversal task sessions in individual experimental groups (WT = wild-type mice, SCA1 = SCA1 mice, 0 = saline, E = edaravone). (**b**) Overall percentage of correct maze arm choices in testing sessions only (both initial learning and reversal task together) in individual experimental groups, where lines represent group-specific prediction from the Bayesian model and its 95% credible intervals. (**c**) The posterior probability distribution for log odds between correct choice percentages in compared groups (in testing sessions only, acquisition sessions are not involved). Curves represent the posterior probability distribution, and lines below the curve show the 95% credible interval for the log odds. The exact values for the estimated log odds and their credible intervals are shown in brown color (top), and the probability of direction is shown in purple color (right).

The water T-maze was used to examine the learning ability of the mice in the initial learning phase of the test, and cognitive and behavioral flexibility in the subsequent reversal task after changing the escape platform position. SCA1 mice achieved worse performance in the testing sessions of both initial learning as well as the reversal task than wild-type mice (probability of direction was 0.993 with log odds of 0.95 in the initial learning phase and 0.996 with log odds of 1.09 in the reversal task; Figure 5). The effect of edaravone was not visible in SCA1 mice (probability of direction was 0.5 and 0.6 in the initial learning and reversal task, respectively). In WT mice, the treatment effect was rather uncertain—the probability of direction was 0.71 and 0.971 (with log odds of 0.82) in the initial learning and reversal task, respectively (Figure 5).

### 2.5. Forced Swimming Test

**Figure 6:**
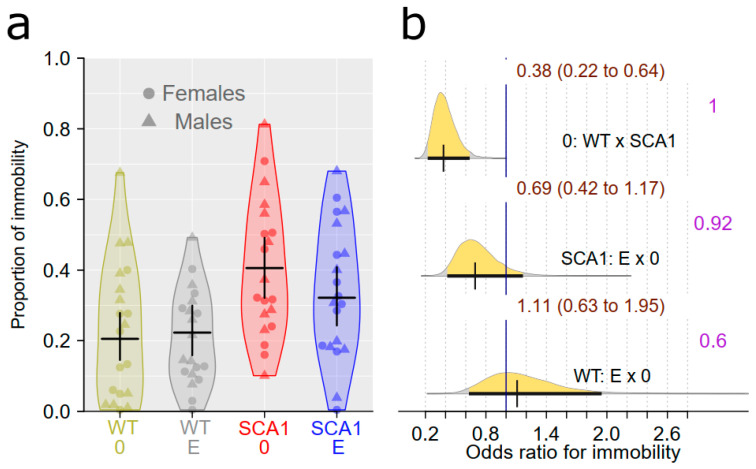
Forced swimming test. (**a**) The proportion of immobility in individual experimental groups (WT = wild-type mice, SCA1 = SCA1 mice, 0 = saline, E = edaravone), where lines represent group-specific prediction from the Bayesian model and its 95% credible intervals. (**b**) The posterior probability distribution for odds ratios (ORs) between immobility values in compared groups. Curves represent the posterior probability distribution, and lines below the curve show the 95% credible interval for the OR. The exact values for the estimated OR and its credible interval are shown in brown color (top), and the probability of direction is shown in purple color (right).

The forced swimming test is intended to show depressive-like behavior or learned helplessness, manifested as immobility responses [47]. Healthy mice had lower immobility in the forced swimming test, with the probability of direction being 1 (odds ratio of 0.38) compared to SCA1 (Figure 6). We did not detect any plausible effect of edaravone (probability of direction was 0.92 with odds ratio of 0.69 in SCA1 and 0.6 in WT; Figure 6).

### 2.6. Muscle Strength Measurement 

**Figure 7:**
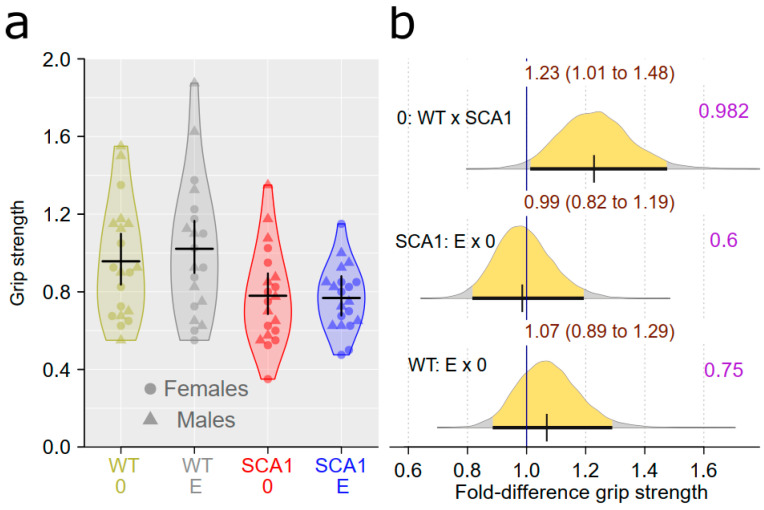
Muscle strength. (**a**) Grip strength in individual experimental groups (WT = wild-type mice, SCA1 = SCA1 mice, 0 = saline, E = edaravone), where lines represent group-specific prediction from the Bayesian model and its 95% credible intervals. (**b**) The posterior probability distribution for fold differences (FDs) between grip strength in compared groups. Curves represent the posterior probability distribution, and lines below the curve show the 95% credible interval for the FD. The exact values for the estimated FD and its credible interval are shown in brown color (top), and the probability of direction is shown in purple color (right).

Muscle strength, measured using the grip strength test, was estimated to be 1.23 (95% CI: 1.01 to 1.48) times greater in WT mice than in SCA1 mice, with the probability of direction of 0.982. The effect of edaravone was uncertain and probably small, with a probability of direction of 0.6 in SCA1 and 0.75 in WT mice (Figure 7).

### 2.7. Rotarod

**Figure 8:**
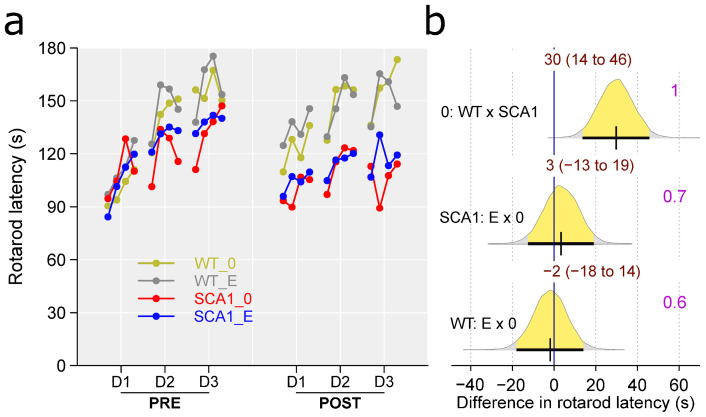
Rotarod. (**a**) Mean fall latencies in individual experimental groups (WT = wild-type mice, SCA1 = SCA1 mice, 0 = saline, E = edaravone) in individual day sessions (D1–D3) of the pre-treatment (PRE) and post-treatment (POST) tests. PRE sessions are used just as covariate for analysis of POST sessions that are of our primary interest. (**b**) The posterior probability distribution for absolute difference between fall latencies in POST sessions in compared groups. Curves represent the posterior probability distribution, and lines below the curve show the 95% credible interval for the difference. The exact values for the estimated difference and its credible interval are shown in brown color (top), and the probability of direction is shown in purple color (right).

The mice were tested on the accelerating rotarod to assess their movement coordination and balance ability. Pre-treatment testing was used to measure the individual’s level of motor skills, serving as a covariate when analyzing genotype and treatment effect in the more advanced stage of the disease in the post-treatment rotarod sessions. SCA1 mice showed significantly shorter fall latencies in the rotarod test, with the probability of direction being 1 and effect size (difference) estimated to be 30 s. The performance of edaravone-treated mice in the rotarod test was similar to that of saline-treated animals. Thus, we did not detect any plausible effect of edaravone (probability of direction was 0.7 in SCA1, 0.6 in WT mice; Figure 8). 

### 2.8. Cerebellar Molecular Layer

**Figure 9:**
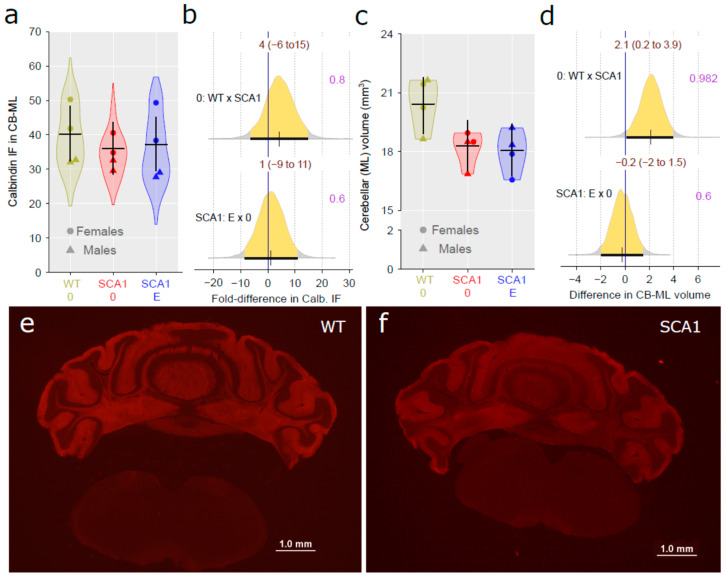
(**a**) Anti-calbindin immunofluorescence signal density in the cerebellar molecular layer and (**c**) cerebellar molecular layer volume in individual experimental groups (WT = wild-type mice, SCA1 = SCA1 mice, 0 = saline, E = edaravone), where lines represent group-specific prediction from the Bayesian model and its 95% credible intervals. (**b**) The posterior probability distribution for fold differences (FDs) between compared groups and (**d**) the posterior probability for absolute differences in anti-calbindin immunofluorescence signal density and molecular layer volume, respectively. Curves represent the posterior probability distribution, and lines below the curve show the 95% credible interval for the effect sizes (FD or absolute difference). The exact values for the estimated effect size and its credible interval are shown in brown color (top), and the probability of direction is shown in purple color (right). (**e**,**f**) Example of anti-calbindin immunofluorescence in the cerebellum of a WT and SCA1 mouse, respectively.

Calbindin was detected immunohistochemically in the cerebellum as a marker of Purkinje cells, including their dendrites distributed in the molecular layer. The volume of the cerebellar molecular layer was estimated. There was only no plausible difference in the density of anti-calbindin immunofluorescence signal in the cerebellar molecular layer between the genotypes (probability of direction was 0.8; Figure 9a,b). However, the cerebellar molecular layer volume was significantly reduced in SCA1 mice compared to the WT cerebellum (probability of direction was 0.982, with estimated difference of 2.1 mm^3^; Figure 9c,d). In any case, edaravone treatment had no apparent effect either on the molecular layer volume or on calbindin density in SCA1 mice (probability of direction was 0.6 for both parameters; Figure 9).

### 2.9. Hippocampus

**Figure 10:**
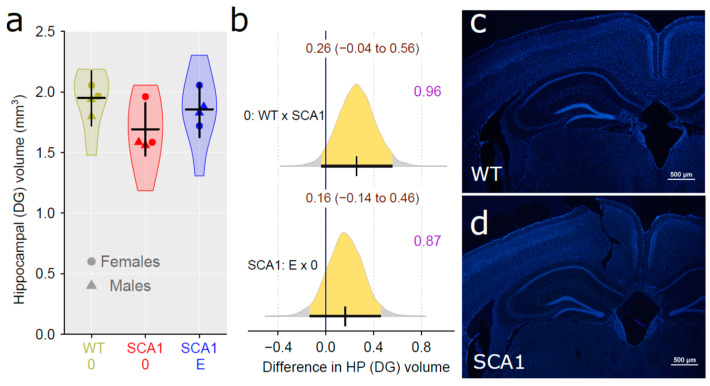
(**a**) Hippocampal dentate gyrus volume in individual experimental groups (WT = wild-type mice, SCA1 = SCA1 mice, 0 = saline, E = edaravone), where lines represent group-specific prediction from the Bayesian model and its 95% credible intervals. (**b**) The posterior probability distribution for absolute differences between dentate gyrus volume in compared groups. Curves represent the posterior probability distribution, and lines below the curve show the 95% credible interval for the difference. The exact values for the estimated difference and its credible interval are shown in brown color (top), and the probability of direction is shown in purple color (right). (**c**,**d**) Example of the hippocampus with nuclei visualized using DAPI in a WT and a SCA1 mouse, respectively.

**Figure 11:**
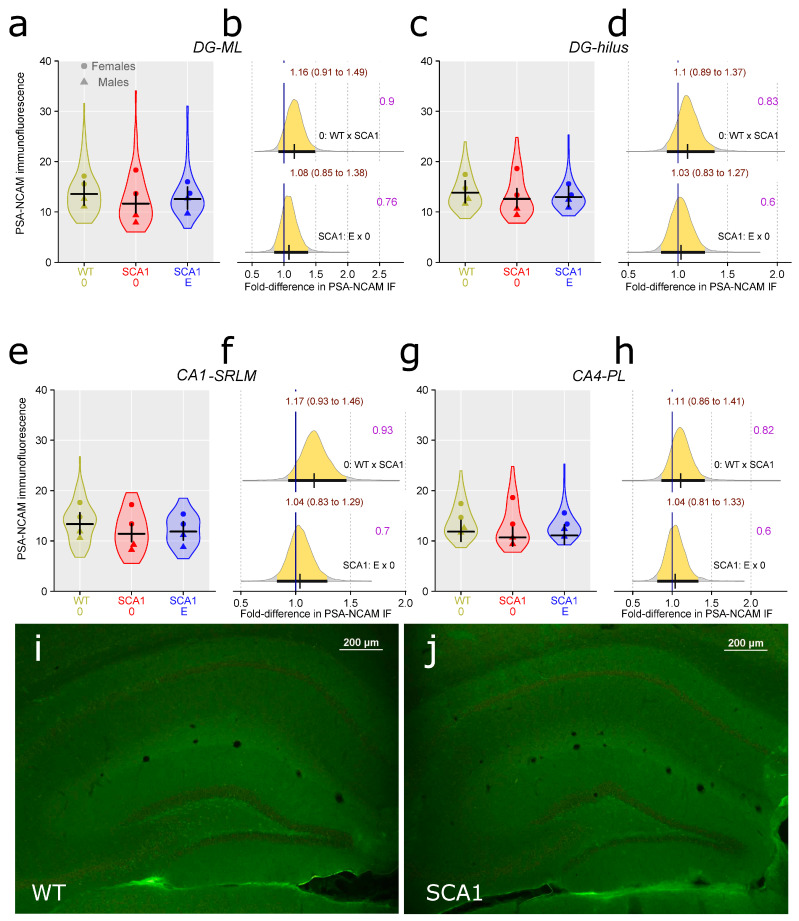
Anti-PSA-NCAM immunofluorescence signal density in the hippocampal regions: (**a**) dentate gyrus molecular layer (DG-ML), (**c**) hilus of the dentate gyrus DG-hilus), (**e**), CA1 stratum lacunosum-moleculare (CA1-SRLM), and (**g**) CA4 pyramidal layer (CA4-PL) in individual experimental groups (WT = wild-type mice, SCA1 = SCA1 mice, 0 = saline, E = edaravone). (**b**,**d**,**f**,**h**) The posterior probability distribution for fold differences (FDs) between compared groups in PSA-NCAM density in the DG-ML, DG-hilus, CA1-SRLM, and CA4-PL, respectively. Curves represent the posterior probability distribution, and lines below the curve show the 95% credible interval for the FD. The exact values for the estimated FD and its credible interval are shown in brown color (top), and the probability of direction is shown in purple color (right). (**i**,**j**) Example of anti-PSA-NCAM immunofluorescence in the hippocampus of a WT and a SCA1 mouse, respectively.

Since hippocampal neuropathology is involved in SCA1 [4,8], the dentate gyrus volume was estimated, using principles of unbiased stereology. We found a rather uncertain reduction in the volume in SCA1 mice as compared with WT mice, although the difference was close to the border of plausibility (probability of direction was 0.96, with difference of 0.26 mm^3^; Figure 10). The effect of edaravone on the dentate gyrus was not obvious (probability of direction was 0.87; Figure 10). Anti-PSA-NCAM (one of the neuroplasticity markers) immunofluorescence examination did not show any plausible decrease in signal density in the hippocampus of SCA1 mice when compared with WT animals (probability of direction was 0.9, with a fold difference of 1.16 for the dentate gyrus molecular layer, 0.83 for hilus of the dentate gyrus, 0.93 with a fold difference of 1.17 for CA1 stratum lacunosum-moleculare, and 0.82 for CA4 pyramidal layer; Figure 11). Furthermore, no apparent effect of edaravone on PSA-NCAM density was detected (probability of direction was 0.76 for the dentate gyrus molecular layer, 0.6 for hilus of the dentate gyrus, 0.7 for CA1 stratum lacunosum-moleculare, and 0.6 for CA4 pyramidal layer; Figure 11).

### 2.10. Examination of Mitochondrial Functions

**Figure 12:**
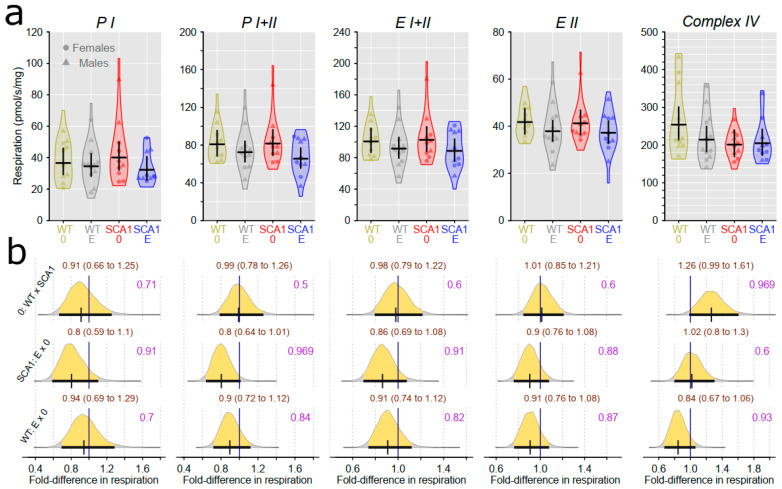
Mitochondrial respiratory states expressed per mg tissue wet weight in the cerebellum. (**a**) Respiratory phosphorylating (PI, PI + II) and electron-transporting (EI + II, EII) capacities, and complex IV activity in individual experimental groups (WT = wild-type mice, SCA1 = SCA1 mice, 0 = saline, E = edaravone), where lines represent group-specific prediction from the Bayesian model and its 95% credible intervals. (**b**) The posterior probability distribution for fold differences (FDs) between mitochondrial respiration in compared groups. Curves represent the posterior probability distribution, and lines below the curve show the 95% credible interval for the FD. The exact values for the estimated FD and its credible interval are shown in brown color (top), and the probability of direction is shown in purple color (right).

**Figure 13:**
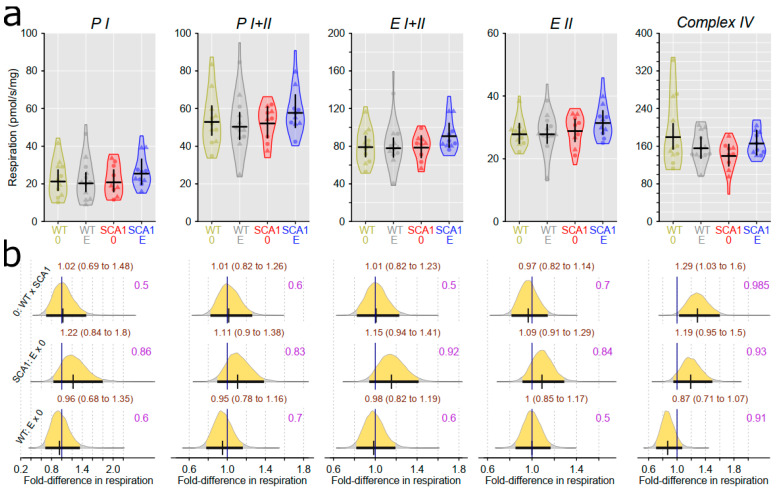
Mitochondrial respiratory states expressed per IU citrate synthase activity in the cerebellum. (**a**) Respiratory phosphorylating (PI, PI + II) and electron-transporting (EI + II, EII) capacities, and complex IV activity in individual experimental groups (WT = wild-type mice, SCA1 = SCA1 mice, 0 = saline, E = edaravone), where lines represent group-specific prediction from the Bayesian model and its 95% credible intervals. (**b**) The posterior probability distribution for fold differences (FDs) between mitochondrial respiration in compared groups. Curves represent the posterior probability distribution, and lines below the curve show the 95% credible interval for the FD. The exact values for the estimated FD and its credible interval are shown in brown color (top), and the probability of direction is shown in purple color (right).

**Figure 14:**
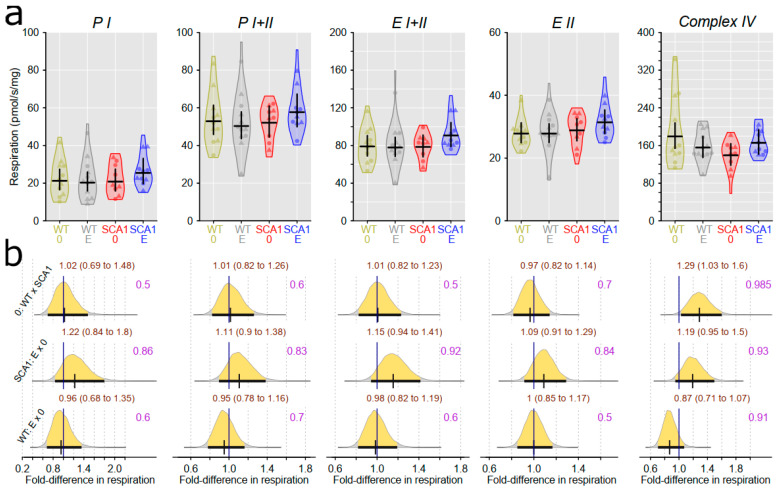
Mitochondrial respiratory states expressed per mg tissue wet weight in the hippocampus. (**a**) Respiratory phosphorylating (PI, PI + II) and electron-transporting (EI + II, EII) capacities, and complex IV activity in individual experimental groups (WT = wild-type mice, SCA1 = SCA1 mice, 0 = saline, E = edaravone), where lines represent group-specific prediction from the Bayesian model and its 95% credible intervals. (**b**) The posterior probability distribution for fold differences (FDs) between mitochondrial respiration in compared groups. Curves represent the posterior probability distribution, and lines below the curve show the 95% credible interval for the FD. The exact values for the estimated FD and its credible interval are shown in brown color (top), and the probability of direction is shown in purple color (right).

**Figure 15:**
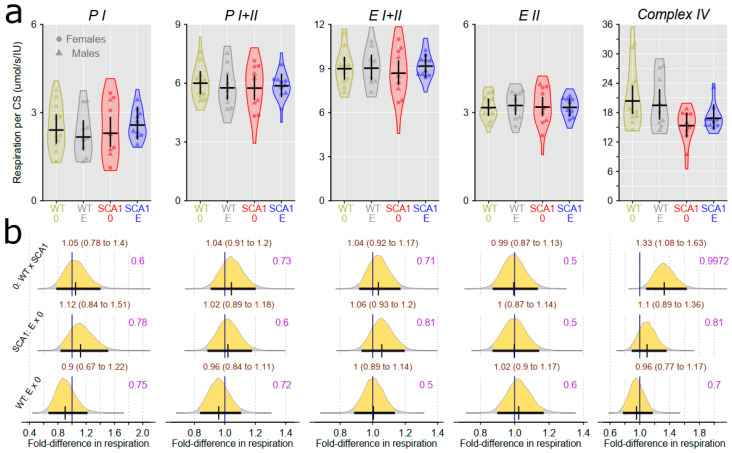
Mitochondrial respiratory states expressed per IU citrate synthase activity in the hippocampus. (**a**) Respiratory phosphorylating (PI, PI + II) and electron-transporting (EI + II, EII) capacities, and complex IV activity in individual experimental groups (WT = wild-type mice, SCA1 = SCA1 mice, 0 = saline, E = edaravone), where lines represent group-specific prediction from the Bayesian model and its 95% credible intervals. (**b**) The posterior probability distribution for fold differences (FDs) between mitochondrial respiration in compared groups. Curves represent the posterior probability distribution, and lines below the curve show the 95% credible interval for the FD. The exact values for the estimated FD and its credible interval are shown in brown color (top), and the probability of direction is shown in purple color (right).

Examination of mitochondrial function showed differences between SCA1 and WT mice, in both the cerebellum and the hippocampus (Figure 12, Figure 13, Figure 14 and Figure 15). In the cerebellum of SCA1 mice, a rather uncertain decrease in complex IV activity expressed per milligram of tissue wet weight compared to WT mice was found (probability of direction was 0.969), with oxygen consumption estimated to be 1.26-fold (95% CI: 0.99 to 1.61) larger in WT mice (Figure 12). For the other respiratory states, differences between SCA1 and WT mice were not apparent (Figure 12). If the mitochondrial oxygen consumption was normalized to citrate synthase activity, models estimated 1.28-fold (95% CI: 1.04 to 1.59) larger complex IV activity in the cerebellum of WT mice as compared with SCA1 cerebellum (probability of direction was 0.988; Figure 13). In the hippocampus, SCA1 mice had reduced complex IV activity in mitochondrial respiration, expressed in both units of tissue wet weight and citrate synthase activity—the probability of direction was 0.985 with a fold difference of 1.29 (95% CI: 1.03 to 1.6) and 0.9972 with a fold difference of 1.33 (95% CI: 1.08 to 1.63), respectively. However, for other states, the differences were not plausible (Figure 14 and Figure 15). No plausible effects of edaravone on mitochondrial function were found either in the cerebellum or in the hippocampus (Figure 12, Figure 13, Figure 14 and Figure 15).

**Figure 16:**
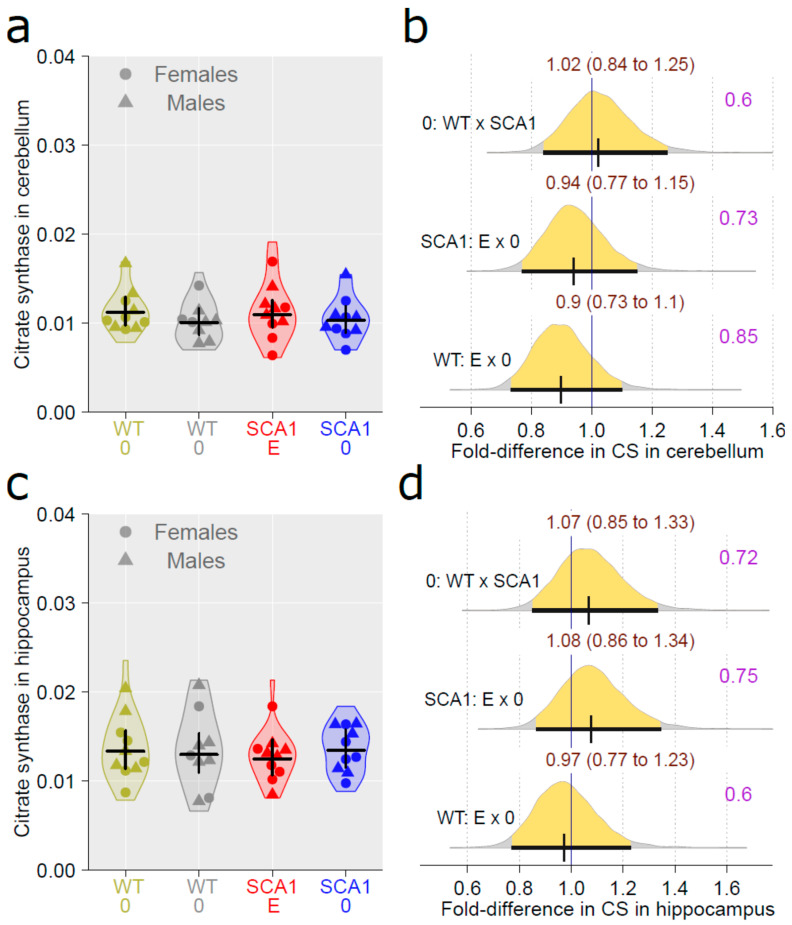
Citrate synthase activity (**a**) in the cerebellum and (**c**) in the hippocampus in individual experimental groups (WT = wild-type mice, SCA1 = SCA1 mice, 0 = saline, E = edaravone), where lines represent group-specific prediction from the Bayesian model and its 95% credible intervals. (**b**,**d**) The posterior probability distribution for fold difference (FD) between citrate synthase activity in compared groups in the cerebellum and in the hippocampus, respectively. Curves represent the posterior probability distribution, and lines below the curve show the 95% credible interval for the OR. The exact values for the estimated FD and its credible interval are shown in brown color (top), and the probability of direction is shown in purple color (right).

In terms of citrate synthase activity, widely used as a marker of mitochondrial density [48], no plausible effect of the genotype (probability of direction 0.6 for the cerebellum and 0.72 for the hippocampus) or of edaravone (for the cerebellum, probability of direction was 0.73 in SCA1 mice and 0.85 in WT mice; for the hippocampus, probability of direction was 0.75 in SCA1 mice and 0.6 in WT mice) was found (Figure 16).

### 2.11. Determination of the Level of Brain-Derived Neurotrophic Factor and the Inflammatory Marker Interleukin 6

**Figure 17:**
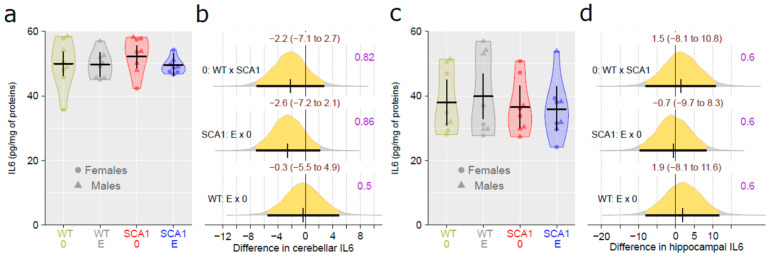
IL6 level in (**a**) the cerebellum and (**c**) the hippocampus in individual experimental groups (WT = wild-type mice, SCA1 = SCA1 mice, 0 = saline, E = edaravone), where lines represent group-specific prediction from the Bayesian model and its 95% credible intervals. (**b**,**d**) The posterior probability distribution for absolute differences between compared groups in cerebellar and hippocampal IL6 levels, respectively. Curves represent the posterior probability distribution, and lines below the curve show the 95% credible interval for the differences. The exact values for the estimated differences and their credible intervals are shown in brown color (top), and the probability of direction is shown in purple color (right).

**Figure 18:**
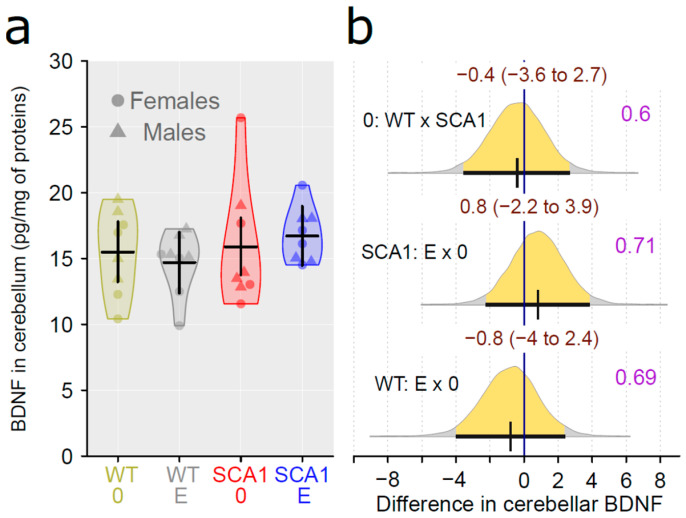
(**a**) Cerebellar BDNF level in individual experimental groups (WT = wild-type mice, SCA1 = SCA1 mice, 0 = saline, E = edaravone), where lines represent group-specific prediction from the Bayesian model and its 95% credible intervals. (**b**) The posterior probability distribution for absolute difference between BDNF levels in compared groups. Curves represent the posterior probability distribution, and lines below the curve show the 95% credible interval for the difference. The exact values for the estimated difference and its credible interval are shown in brown color (top), and the probability of direction is shown in purple color (right).

Interleukin 6 (IL6) level was measured in the cerebellum and hippocampus, using enzyme-linked immunosorbent assay (ELISA) and adjusted to protein level. There were no plausible differences in IL6 level between SCA1 and WT mice, either in the cerebellum or the hippocampus (probability of direction was 0.82 and 0.6, respectively; Figure 17). In addition, no plausible effects of edaravone on IL6 levels were found (for the cerebellum, probability of direction was 0.86 in SCA1 mice and 0.5 in WT mice; for the hippocampus, probability of direction was 0.6 in both SCA1 and WT mice; Figure 17). The brain-derived neurotrophic factor (BDNF) was measured in the cerebellum only, because the quantity of hippocampal tissue was not sufficient for both IL6 and BDNF measurements. Cerebellar BDNF levels were similar in SCA1 and WT mice (probability of direction was 0.6). In addition, the effect of edaravone was not apparent (probability of direction was 0.71 in SCA1 mice and 0.69 in WT mice; Figure 18).

## 3. Discussion

This study aimed to examine the potential effects of the mitochondria-targeting drug edaravone in a mouse model of SCA1. The study also included examinations of wild-type mice of the same strain and colony as indicators of basal levels of the measured parameters and to see possible negative or positive effects of edaravone in healthy individuals. Therefore, the experiment also permitted a comparison of saline-treated SCA1 and healthy mice, to verify or refine earlier descriptions of the pathological phenotype of this mouse model. We confirmed most of the behavioral, cognitive, and motor deficits previously described in SCA1 mice [4,6].

SCA1 mice showed reduced activity in the open field, but not significantly increased preference for the arena periphery. Even when thigmotaxis was expressed as a percentage of time spent in the peripheral zone instead of a percentage of distance moved, the difference was not apparent. An increase in immobility responses in the forced swimming test and reduced learning ability and cognitive flexibility in the T-maze were observed as expected. The slightly higher percentage of correct responses in the first sessions of the reversal task in the T-maze in SCA1 mice than in WT animals seen in Figure 5 was probably only due to the overall worse performance of SCA1 mice in the initial learning phase of the test. After changing the platform position, incorrect choices became correct and thus increased the score of the SCA1 mice. On the other hand, WT mice followed the former well-learned lateralization for the few trials needed to learn a new state. In fact, this phenomenon confirms the good learning ability of WT mice and the cognitive deficit in SCA1 mice. Although 23- to 24-week-old SCA1 mice are not apparently ataxic, motor tests showed deficits in muscle strength, balance and movement coordination (rotarod test), and overall gait structure (CatWalk).

Among behavioral examinations, an exception was the sucrose preference test, in which reduced sucrose consumption in 12-week-old SCA1 mice was found previously [4], but not in the present study, which used much older animals. Since hippocampal pathology precedes the development of significant cerebellar dysfunction [4] and, as Tichanek [7] pointed out, cerebellar impairment can induce opposite changes in behavior compared to hippocampal dysfunction, some behavioral abnormalities can be reduced with age and progress of the cerebellar component of the disease. Furthermore, the results of this test are controversial in general, since both reduced and increased sucrose preference in SCA1 mice have been previously reported [4,23]. In cerebellar mutant mice, depressive- and anxiety-like behavior and anhedonia on one hand and reduced anxiety on the other hand can be manifested. The first occurs in the case of significant extracerebellar neuropathology, the latter rather in mice with selective cerebellar damage [4,23,49,50,51,52,53,54,55]. 

SCA1 mice had a smaller volume of the cerebellar molecular layer, probably in part due to a reduction in Purkinje cell dendrites [56]. Unexpectedly, only an uncertain decline of calbindin immunoreactivity in the molecular layer of the cerebellum was found. We can speculate that loss of calbindin expression and/or dendritic arborization might be nearly proportional to the volume loss at this stage of the disease, maintaining calbindin density close to the normal level. We did not find any abnormal BDNF levels in the cerebellum of SCA1 mice. Nevertheless, the changes in BDNF described by others were age-dependent and found in another SCA1 mouse strain [57]. Contrary to our previous findings [4], hippocampal dentate gyrus volume reduction and neuroplasticity marker PSA-NCAM density in SCA1 mice were rather uncertain, but cannot be negated completely by the present study.

Our study confirmed mitochondrial dysfunction in the hippocampus of SCA1 mice at the age of 23 weeks. However, its extent was somehow less expressed than at the age of 11–13 weeks, being limited to the reduced activity of complex IV [4]. In addition, complex IV activity was also decreased in the cerebellum of SCA1 mice, which was not yet apparent in the younger age category [4]. This finding provides further evidence that hippocampal pathology precedes the onset of cerebellar changes in SCA1^154Q/2Q^ mice. Complex IV, cytochrome c oxidase, is the terminal electron-transferring complex of the mitochondrial respiratory system that utilizes four protons to reduce dioxygen to water. Its dysfunction or dysregulation is considered a promising marker of various neurodegenerative diseases [58], although its role in the pathophysiology of cerebellar ataxia is not yet well understood. In a *Coq8a*^−/−^ constitutive knockout mouse model of autosomal recessive ataxia type 2 with progressive cerebellar ataxia, exercise intolerance, and memory impairment, mitochondria seem to belong to culprits of the whole pathological process: Purkinje neurons display altered expression of respiratory complexes, in particular complex IV, at presymptomatic stages of the disease [59]. However, the *COQ8A* gene is directly involved in the production of coenzyme Q10, playing an essential role in oxidative phosphorylation, whereas the role of ataxin 1 in the mitochondrial bioenergetics seems to be more complex and bidirectional: In *Atxn1*-KO knockout mice at the age of 5 weeks, spectrophotometric activities of individual respiratory complexes in the isolated cerebellar mitochondria displayed decreased (complex I), increased (complex II, complex III), and unchanged (complex IV) values compared to WT mice [2]. In addition, the age of the experimental animals could also have a substantial impact on the mitochondrial functional parameters, since at younger ages, mitochondrial respiratory states PI and PI+II in the hippocampus tended to increase between the ages of 4 and 11 weeks [60]. At 6 months of age, expressions of mitochondria-related genes encoding complex IV, creb-1, β-AMPK, and Tfam in the brain of NMRI mice were reported to be significantly elevated, compared to 3-month-old animals [61]. Thus, it cannot be excluded that a similar trend of mitochondrial changes from juvenile to young adult age was slowed down in our SCA1^154Q/2Q^ mice and that the only significant mitochondrial respiratory dysfunction in the symptomatic stage of the disease is related to the complex IV dysfunction.

In general, we can conclude that SCA1^154Q/2Q^ had a significant pathological phenotype at the age of 23–24 weeks and that there are many parameters on the motor, cognitive, behavioral, cellular, as well as biochemical levels to be ameliorated by efficient therapy in this preclinical model. However, we did not see any clinically significant improvement in edaravone-treated SCA1 mice. In addition, edaravone had no adverse effects, either in SCA1 or in healthy mice.

Edaravone is a neuroprotective drug, acting as a free radical scavenger. In experimental or clinical studies, it has been shown to have positive effects in diverse neurological pathological conditions, e.g., ischemic cerebral stroke, ALS, traumatic spinal injury, and Alzheimer’s disease [35,36,62,63,64]. It also aids in diseases of other organ systems [65,66]. In clinical praxis, edaravone is approved and used for the therapy of acute ischemic stroke and ALS [37,64]. Based on the mechanisms of its effects and known therapeutic effects, edaravone appeared as a good candidate to be tested in hereditary neurodegenerative ataxias. In SCA1 and particularly in SCA1^154Q/2Q^ mice as a model of this disease, mitochondrial dysfunction participates in the disease pathogenesis [4,45]. Despite such predispositions, edaravone failed in the treatment of SCA1 in the preclinical model. There are many potential explanations for the absence of a significant therapeutic effect of edaravone. 

Firstly, we should consider the possibility that edaravone does not target the pathogenetic mechanism essential for SCA1 progress. We can, for instance, speculate that oxidative stress and mitochondrial dysfunction are not the underlying mechanisms of cell dysfunction and degeneration in SCA1. If this assumption is true, edaravone as a scavenger would not cure SCA1, but could only reduce the additional minor impacts of oxidative stress occurring just as a secondary or collateral phenomenon accompanying primary neuropathology. Nevertheless, oxidative stress is considered to be an important pathogenic mechanism in SCA1 [45,67].

There is also the question of edaravone dosage. In small animals, such as mice, dosage per 1 kg of the body weight must often be higher than in humans, because of the relatively more intensive metabolism. However, we used a daily dose of 40 mg/kg, which is much higher than that used in humans [68,69]. Furthermore, our dose was comparable to, or even slightly higher than, the oral dosage shown as being effective in a rat model of cerebral artery occlusion [70] or in a mouse model of Alzheimer’s disease [62]. Administration of edaravone suspension directly into the mouths of the mice allowed relatively good control over the drug intake. On the other hand, the biological availability of any orally administered drug is always uncertain, because resorption depends on many factors that cannot be simply controlled. However, the same problem exists in any *peroral* drug administration in human patients as well. In humans, the bioavailability of edaravone administered *per os* is about 60% [68]. In mice, the bioavailability of orally delivered edaravone was estimated as 38% of the intravenous administration [62].

SCA1 is a progressive neurodegeneration. To rescue the neurons and maintain substantial cerebellar reserve, therapy should start before substantial neuronal loss has developed [71,72]. In our study, therapy started at the age of 8 weeks, i.e., in the early disease stage, with just mild and partial symptoms and minimum neuropathology [4,23]. Indeed, irreversible processes starting before clinical manifestation and limiting the effect of further therapy cannot be excluded. However, a study by Zu et al. [73] provided evidence that if the expression of mutant ataxin 1 was stopped in the early disease stages, the manifestation of SCA1 was fully reversible in conditional mutants, whereas, after halting the expression in a more advanced stage, only partial recovery was achieved. Similar reversibility of symptoms has been reported in a mouse model of SCA3 [74]. The sooner the therapy starts, the better. However, mice in our study were treated at the age at which highly efficient therapy should still be manifested at least by a delay of disease progress and thereby by better performance in treated mice as compared with the control group. 

Our study was a classic drug repurposing approach. Drug repurposing is based on the expectation that a medicament effective in the treatment of a certain disease or certain diseases could also have a therapeutic effect in other diseases having similar etiology and/or pathogenesis. In many neurological diseases, neuroprotective, antioxidant, and plasticity-supporting substances can have a non-specific therapeutic effect. In a drug repurposing approach, animal model-based and other preclinical studies are one of the first steps to verify whether the proposed drug modifies a respective disease course and symptoms and to investigate the mechanisms employed in therapeutic effects in a particular disease, before realizing more practically and ethically problematic clinical trials.

Unfortunately, the studies in animals and human patients do not always provide the same results, and the suggested drug is not always effective in the newly proposed indication. For example, riluzole had no acute effect in SCA1 mice [75], and long-lasting therapy even promoted neuropathology in SCA3 mice [76]. In human patients with hereditary ataxias, riluzole has been suggested as being effective but offered only a moderate reduction in the Scale for the Assessment and Rating of Ataxia (SARA) score [77]. Similarly, lithium has not fulfilled the expectations. In SCA1 mice, lithium therapy improved neural functions [8], but it failed in the SCA3 mouse model and patients [78,79]. These findings also suggest that the effects can be disease-specific, even in the frame of the group of spinocerebellar ataxias.

Several studies on preclinical models showed potential therapies for SCA1, e.g., block of ataxin 1 expression in double mutants [73], use of antisense oligonucleotides [80,81], enzymatic cleavage of CAG repeat RNA [82], or neurotransplantation [83]. However, for SCA1, these approaches are rather in the stage of experimental research and relatively invasive, and safety for human patients needs to be assessed. Simple pharmacotherapy with approved efficiency, acceptable safety, and the capability to halt the degenerative process is still missing for SCA1.

## 4. Materials and Methods

### 4.1. Animals

Heterozygous knock-in mice with 154 CAG repeats within exon 8 of the *ATXN1* gene (SCA1^154Q/2Q^) of the B6.129S-*Atxn1^tm1Hzo^*/J strain (Jackson Laboratory) [6] were used for this study. In these mice, both cerebellar and hippocampal neuropathology develops, and they show motor, cognitive, and behavioral abnormalities well characterizing the SCA1 phenotype [4,6]. Healthy mice with a normal number of CAG repeats (SCA1^2Q/2Q^) from the same colony (i.e., wild type, WT, littermates) were used as controls. The mice were kept under standard laboratory conditions at a temperature of 22–24 °C and relative humidity of 30–60% with a 12:12 h light/dark cycle (light period from 6 a.m. to 6 p.m.). The experiments were performed during the light period. Commercial pellet diet and water were available ad libitum. Mice were housed individually in plastic boxes with metal lids to eliminate non-standardizable stress from diverse social interaction and potential aggressivity between animals if housed in one cage. 

### 4.2. Design of the Experiment

The treatment with edaravone or saline started at the age of 8 weeks, i.e., before ataxia is detectable [4,23]. Four experimental groups of mice were established: SCA1^154Q/2Q^ edaravone-treated (SCA1_E), SCA1^154Q/2Q^ saline-treated (SCA1_0), SCA1^2Q/2Q^ edaravone-treated (WT_E), and SCA1^2Q/2Q^ saline-treated (WT_0). Each experimental group consisted of two cohorts of mice handled using different protocols (Figure 1). Mice of cohort *a* (SCA1_E: 10 males, 10 females; SCA1_0: 10 males, 10 females; WT_E: 10 males, 10 females; WT_0: 11 males, 9 females) were treated for 95 days and then subjected to a series of motor, cognitive, and behavioral tests performed during the next two weeks. Treatment with edaravone or saline continued during these two weeks, until the day of the last functional test. On the day following the completion of the series of functional tests, the mice were euthanized to take brain samples for histological examination (2 males and 2 females per group from both SCA1 groups and from the group WT_0 used as an indicator of normal state). Mice of cohort *b* (SCA1_E: 5 males, 5 females; SCA1_0: 5 males, 5 females; WT_E: 7 males, 5 females; WT_0: 5 males, 5 females) were euthanized after 100 days of edaravone or saline treatment without any further experimental manipulations. The brains of all mice of cohort *b* were used for examination of mitochondrial functions, and the brains of 4 males and 4 females per experimental group were also used for biochemical examination of IL 6 and BDNF using the ELISA method (see Section 4.5 for details on brain structure preparation).

### 4.3. Treatment

Edaravone (3-methyl-1-phenyl-2-pyrazoline-5-one, M70800, Sigma-Aldrich, Saint Louis, MO, USA) in a dose of 40 mg per kg of the body weight in saline (dilution of 50 mg per ml) was administered perorally (injected with a pipette into the mouth of the mouse while checking for possible liquid leakage) daily from the 8th week of age for 108 days (cohort *a*, for the last 13 days simultaneously with functional tests) or for 100 days (cohort *b*). An adequate volume of saline was administered to the control mice (SCA1_0, WT_0). 

### 4.4. Motor, Cognitive, and Behavioral Tests

#### 4.4.1. Sucrose Preference Test

The degree of depression-like behavior or anhedonia was examined by the sucrose preference test. Two bottles were placed in the mouse cage—one with tap water, the other containing 3% sucrose solution. Positions of the bottles were switched daily to minimize the potential effect of place preference. The intake of tap water and sucrose solution was measured daily for 4 days. The consumption was added up over 4 days of the test. Sucrose preference was expressed as relative sucrose consumption over the total fluid intake.

#### 4.4.2. Open Field Test

Spontaneous activity (novel environment exploration) was monitored in the open field. A white plastic arena sized 50 cm × 50 cm × 50 cm and illuminated by diffuse light (250–300 lux) was used. The mouse was placed into the center of the arena and left undisturbed for 10 min to explore freely. Its movement was recorded by the EthoVision XT14 tracking system (Noldus Information Technology, Wageningen, The Netherlands). The total distance moved and thigmotaxis (the portion of the length of trajectory realized in a peripheral zone along the arena walls of a width of 6 cm) were analyzed.

#### 4.4.3. Gait Analysis

The gait was examined using the CatWalk XT10.6 system (Noldus Information Technology, The Netherlands) [84] which has been shown to display gait disturbances in ataxic mice [85]. Five runs were analyzed for each mouse, and parameter values were averaged. A total of 53 parameters, which included mean values and their standard deviations, were monitored. Such a large number of parameters was viewed as a complex (see Section 4.9), and the parameters were divided into three groups according to their correlations. For a complete overview of the evaluated parameters, see Supplementary Material S1.

#### 4.4.4. Water T-Maze

Memory and cognitive flexibility were examined in the water T-maze. The arms of the T-shaped arena were 7 cm wide; two of them were 30 cm long, and one (the starting arm) was 38 cm long. The maze was filled with opaque water (with non-toxic white food coloring) at the temperature of 21–23 °C. The mice learned to navigate to the platform, which was hidden 0.5 cm under the water surface. The location of the hidden platform in one of the two arms varied during the 3-day (D1–D3) testing scheme, with 4 sessions (S1–S4) per day and 4 trials per session. The trials in one session followed immediately after each other. Between the sessions, the mice had at least 5 min resting periods spent in their home cages. In each trial, the mouse had a maximum of 60 s to find the platform. If not succeeding, it was guided there by the experimenter. After each trial, the mouse was left on the platform for 20 s. In all sessions of D1 and S1 and S2 of D2, the platform was located in the left arm (initial learning). Then, the position of the platform was changed to the right for S3 and S4 of D2 and all sessions of D3 (reversal task). Performance in the test was evaluated by scoring. If the mouse turned well and swam to the platform on the first attempt, it gained 1 point. If its first turn was to the wrong arm (i.e., without the platform) or it did not reach the platform within 60 s, it gained no points. Points from all trials in the session were added up and expressed in percentages. Four points represented 100% success. If the mice demonstrated immobile behavior, they were motivated to move via noise or the gentle touching of the tail, if necessary. 

#### 4.4.5. Forced Swimming Test

Depressive-like behavior or learned helplessness behavior were examined, using the forced swimming test [47]. The mouse was left in the glass cylindrical vessel (16 cm in diameter) filled up to 10 cm below the rim (the mouse did not touch the bottom of the vessel) with water of 26–28 °C for 8 min. Its movement was registered by EthoVision XT14 (Noldus Information Technology, the Netherlands). Time spent in the immobile state was measured, and the results were expressed as relative values to the total duration of the test.

#### 4.4.6. Muscle Strength Measurement

Muscle strength was measured using the grip strength test (Bioseb) on the first day of rotarod testing, before the first rotarod trial (see Section 4.4.7). The mouse was allowed to grasp a metal grid with its forepaws. When the tail was pulled, the force at which the mouse released the grid was measured. The measurement was repeated four times, and the values were averaged.

#### 4.4.7. Rotarod

Motor coordination was examined, using the accelerating rotarod (RotaRod Advanced, TSE Systems GmbH, Berlin, Germany). The examination took place during the 3 days before the start of treatment (PRE) and the last 3 days of treatment (POST). The rod was 4 cm in diameter and 8 cm long. The rotation speed was gradually increased from 0 to 60 rotations per minute (RPM) over a period of 6 min. Fall latency was measured. Four trials were performed daily. The time between the starts of the trials was 16 min. The mean fall latencies were calculated. The rotarod test was repeated for 3 consecutive days. 

### 4.5. Sample Collection

The mice of cohort *a* were euthanized by an overdose of thiopental and transcardially perfused with Ringer’s solution and phosphate-buffered (pH 7.4) 4% paraformaldehyde (PFA) in saline. The brains were removed and left in PFA for 2 h for post-fixation and then in 30% sucrose for cryoprotection overnight. Finally, they were frozen and stored at −80 °C until further histological processing (see Section 4.6).

The mice of cohort *b* were euthanized via cervical dislocation on the next day after completing 100 days of treatment, and the brains were removed. One-half of the cerebellum and one hippocampus were dissected and immediately used to examine mitochondrial functions (see Section 4.7). The second half of the cerebellum and the second hippocampus were stored at −80 °C until further processing, for measuring BDNF and IL6 levels using ELISA (see Section 4.8). 

### 4.6. Histological Examination

The brains were cryo-sectioned into 40 µm frontal slices. Free-floating sections containing the cerebellum (sampling: every 5th section) and the hippocampus (sampling: every 6th section) were processed for immunofluorescent detection of calbindin and PSA-NCAM, respectively. The slices were washed with 0.01 M PBS four times for 5 min, blocked with 10% normal goat serum for 1 h, and then were incubated with primary antibodies (Anti-calbindin, 1:1500, PA5-85669, Invitrogen, Waltham, MA, USA; Anti-PSA-NCAM, 1:500, 14-9118-82, Invitrogen) overnight at room temperature. Thereafter, the slices were rinsed in 0.01 M PBS for 5, 10, and 30 min and incubated with the secondary antibodies (Alexa Fluor 488-goat anti-mouse IgG preadsorbed, 1:400, ab150117, Abcam, Cambridge, UK; Alexa Fluor 594-goat anti-rabbit IgG preadsorbed, 1:400, ab150084, Abcam) for 2 h in the dark at room temperature. Finally, the sections were rinsed in 0.01 M PBS for 5, 10, and 30 min and mounted using Fluoroshield with DAPI (Sigma-Aldrich).

The specimens were visualized using a fluorescent Olympus BX51 microscope (Olympus Corporation, Tokyo, Japan). Photo documentation was performed using a DP70 digital camera microscope (Olympus Corporation, Japan) under standardized exposure time, sensitivity, and excitation intensity. The images were analyzed using Fiji software (ImageJ 1.53t).

The volume of the cerebellar molecular layer and the hippocampal dentate gyrus was estimated using the point grid method and the Cavalieri principle [86,87]. Images showing DAPI staining (cell nuclei labeling) were used for hippocampal dentate gyrus estimation. Cerebellar molecular layer volume was estimated using images showing anti-calbindin immunofluorescence, precisely indicating the Purkinje cell layer as a borderline.

The density of calbindin in the cerebellar molecular layer was analyzed by measuring the color intensity in the molecular layer. The intensity was measured in each slice in 2 randomly spaced samples in the vermis and 4 randomly spaced samples in the cerebellar hemispheres (2 samples in each hemisphere). The density of PSA-NCAM was analyzed by measuring the immunofluorescence signal intensity in the hippocampal regions of the molecular layer of the dentate gyrus, the polymorphic layer of the dentate gyrus hilar region, the pyramidal layer of an area that is labeled as the CA4 subfield by some authors [88], and the stratum lacunosum-moleculare of the CA1 (for further information, see [4]). 

### 4.7. Examination of Mitochondrial Functions

The mitochondrial functions were examined by mitochondrial high-resolution respirometry in the tissue of the cerebellum and the hippocampus of cohort *b*. All measurements were performed in quadruplicate (4× per mouse and brain structure). The dissected tissue was gently dried on a filter paper, weighed, and subsequently homogenized on ice in MiR05 [89] respiration medium, using a PBI-Shredder O2k-Set (Oroboros Instruments, Innsbruck, Austria). Oxygraphy was performed as described elsewhere [4]. Briefly, homogenized tissue samples were inserted into 4 precalibrated oxygraphs (Oroboros, Innsbruck, Austria), each containing 2 separate chambers. After the O_2_ signal was stabilized, the chambers were closed and the standard Substrate-Uncoupler-Inhibitor Titration protocol (SUIT) was applied [90]. NADH-linked substrates, malate (5 mmol/L), glutamate (10 mmol/L), and pyruvate (5 mmol/L) were added to reach the LEAK (L) state, and then ADP (5 mmol/L) was injected to convert the respiration into the OXPHOS (ATP-producing) state. The addition of cytochrome c (10 µmol/L) served to test the integrity of the inner mitochondrial membrane (PI). Thereafter, succinate (50 mmol/L), a complex II substrate, was added to initiate the phosphorylating PI+II state. Stepwise titration of FCCP (0.5 µmol/L titrations) was used to uncouple respiration and phosphorylation and to reach maximal electron transport system capacity (state EI+II). Titration of rotenone (0.5 µmol/L), a complex I inhibitor, helped to evaluate uncoupled complex II capacity (EII), and antimycin A (complex III inhibitor; 2.5 μg/mL) was used to obtain residual oxygen consumption (ROX). Complex IV activity was measured by adding ascorbate (2 mmol/L) and TMPD (0.5 mmol/L; artificial substrate of complex IV), followed by sodium azide (100 mmol/L)—a complex IV inhibitor (to obtain background autooxidation for data correction). All data were corrected for ROX. All samples were kept at −80 °C for later measurement of citrate synthase activity. Oxygen consumption in the individual respiratory states was expressed in pmol O_2_/s/mg tissue wet weight.

The citrate synthase activity was measured to estimate mitochondrial content in the samples from each oxygraph chamber. The assay medium (900 µL; 0.1 mmol/L 5,5-dithio-bis- (2-nitrobenzoic) acid, 0.25% triton-X, 0.5 mmol/L oxaloacetate, 0.31 mmol/L acetyl coenzyme A, 5 µmol/L EDTA, 5 mmol/L triethanolamine hydrochloride, and 0.1 mol/L tris-HCl, pH 8.1) was mixed with 100 µL of the mixed and homogenized chamber content. The rate of absorbance change was measured spectrophotometrically at 412 nm and 30 °C over 200 s. Citrate synthase activity was expressed in IU/g tissue wet weight.

### 4.8. Determination of the Level of BDNF and the Inflammatory Marker IL6

The levels of BDNF in the cerebellum and IL6 in the cerebellum and hippocampus were determined by ELISA. The Human BDNF SimpleStep ELISA kit (with mouse reactivity; ab212166, Abcam) and the Mouse IL6 SimpleStep ELISA kit (ab222503, Abcam, Cambridge, UK) were used according to the manufacturer´s instructions. The absorbance was measured using a Tecan Infinite M200 Pro microplate reader. Data were normalized to the protein level measured using the Bicinchoninic Acid Kit for Protein Determination (BCA1, Sigma-Aldrich, Saint Louis, MO, USA) according to the manufacturer’s instructions. All specimens were processed in duplicate, and the values were averaged.

### 4.9. Statistics

Data analysis was performed using R [91] in R-studio [92]. Statistical models were fitted in a Bayesian framework via the “brms” [93,94] R package v. 3.6.0, employing “Stan” as backhand software for No-U-Turn Sampler (NUTS) probabilistic sampling [95,96]. Two chains, each consisting of 8000 iterations (2000 warm-ups), were used to sample from probability space. The described R code and data will be available on the day of publication at https://github.com/filip-tichanek/edaravonSCA1, accessed on 20 June 2023.

Continuous data of proportions (values from 0 to 1) were analyzed via beta regression with logit link. The proportions of successes/failures (from T-maze) were modeled with a beta-binomial model with logit link. Non-negative right-skewed data were modeled via Gamma regression with log link. Data with approximately Gaussian residuals were fitted with Gaussian models or Student t distribution when there were outliers. All models were checked via posterior predictive check (PPC) [97]. If preliminary visual inspection indicated that sex may noticeably affect the result, we also fitted an alternative model that included sex as a covariate and compared them with each other via leave-one-out cross-validation (LOO) [98]. Longitudinal and otherwise correlated data were analyzed in the same way, but the models included random effects accounting for the dependency.

For the analysis of CatWalk, we originally evaluated 53 gait parameters. As 4 parameters showed many zero values, these were omitted from the analysis. Gait parameters of consistency (standard deviations (SD) of gait parameters) were log-transformed, whereas parameters in percentages were transformed to raw proportions (0–1) and logit-transformed. Given such a high number of parameters, the gait was analyzed as a conglomerate of outcomes with multivariate statistical approaches. At first, we Z-standardized all gait parameters, extracted principal components (PCs) and used the first 3 of them (explaining together 69% of the variability in the gait parameters) as outcomes in a multivariate Bayesian regression model. As the walking speed may strongly affect numerous parameters, the analysis was repeated, but with adjustment for walk speed. As an additional method, we also used permutational analysis of variance (PERMANOVA) with Euclidian distances via “vegan” R package [99].

We used the Gaussian Bayesian mixed-effect model with rotarod latency as an outcome (no averaging) and with the following predictors: day, session, group, day: session, day: group, rot_lat_PRE (mean rotarod latency before the start of treatment from days 2 and 3). 

We used a Bayesian generalized linear model with beta-binomial distribution to model success in the T-maze.

(1) Analysis of overall success from all testing sessions (sessions 4–6 and 11–12).
(2) Analysis of initial learning: sessions 4–6
(3) Analysis of flexibility: sessions 11–12. As the performance may be paradoxically increased in non-learners, the performance in session 7 was included as a covariate in the model. Such a model showed better predictive accuracy, as indicated by leave-one-out cross-validation.

PSA-NCAM immunofluorescence (PSA-NCAM IF) in the hippocampus was analyzed via a Bayesian hierarchical generalized additive model with Gamma distribution and random-intercept effect, represented by mouse ID. As the PSA-NCAM IF showed an apparent sex-related difference, further confirmed with leave-one-out cross-validation, the sex factor was included in the final model. As the PSA-NCAM IF varied across slices from the frontal to caudal regions, the order of slices was included as a factor with a non-linear effect, fitted with thin-plate splines limited to 3 knots. 

Calbindin immunofluorescence intensity was evaluated with Bayesian hierarchical regression, with two levels of random intercept: mouse ID and slice ID (nested in mouse). The model was adjusted for subregion (vermis vs. hemisphere) and immunofluorescence intensity in the neighboring granular layer. 

Hippocampal volumes (volumes of each of the two hippocampi constituted a separate data input) were modeled with a robust regression (Student t-distribution), with mouse ID as a random-intercept factor.

For Bayesian models, we used Gaussian priors for all fixed-effect parameters (including the intercept) and default priors otherwise. For the effect of edaravone, we used a prior with zero mean and sigma of 1.2 (or 1.2*SD of SCA1 data in case of models with Gaussian or Student-t distribution). For the genotype effect, we used a weakly informative prior, utilizing the information from our previous publication [4] when methodologies were comparable. See https://github.com/filip-tichanek/edaravonSCA1, accessed on 20 June 2023 for more details.

Effect sizes are shown in absolute effect (Gaussian models), odds ratio (OR; for beta distribution), log(OR) (log odds, used for T-maze due to long tails of the posterior distribution), and fold difference/change (FD) (for Gamma models), indicating how many times larger the measure is in one group compared to the other. For all the effects of interest, we show the whole posterior probability distribution. FD and OR have zero effect at 1; absolute effect and log(OR) have zero effect at 0. We used 95% credible intervals to report uncertainty about the estimated effects and also calculated the probability of direction which may be interpreted as an index (0.5 to 1) representing the certainty that the effect goes in a particular direction [100]. For PERMANOVA, as a non-Bayesian analysis, the *p*-value is reported. Generally, the probability of direction > 0.975 and *p*-value < 0.05 were considered as an indication of the plausible effect. Otherwise, the effect was described as an effect with an uncertain direction or simply “uncertain”.

## 5. Conclusions

Despite the promising results of previous studies pointing out the therapeutic potential of edaravone as a treatment for diverse neurological diseases [34,35,36], we found only highly uncertain treatment effects of edaravone for all outcomes. Thus, our results do not represent any support for the therapeutic potential of edaravone in SCA1. On the other hand, we have confirmed most of the functional deficits and neuropathological traits of SCA1 mice [4,6].

## Data Availability

The data presented in this study are available at https://github.com/filip-tichanek/edaravonSCA1, accessed on 20 June 2023.

## Supplementary Materials

The following supporting information can be downloaded at: https://www.mdpi.com/article/10.3390/ijms241310689/s1.

## Abbreviations

ALS	amyotrophic lateral sclerosis
BCA	bicinchoninic acid
BDNF	brain-derived neurotrophic factor
ELISA	enzyme-linked immunosorbent assay
FD	fold difference
IL6	interleukin 6
OR	odds ratio
PC	principal component
ROS	reactive oxygen species
ROX	residual oxygen consumption
RPM	rotation per minute
SCA1	spinocerebellar ataxia type 1
SCAs	spinocerebellar ataxias
WT	wild type
