# Supplementary material for: Experimental Treatment with Edaravone in a Mouse Model of Spinocerebellar Ataxia 1
Source: Int J Mol Sci. 2023 Jun 26;24(13):10689. doi: 10.3390/ijms241310689 (PMC10341848; doi:10.3390/ijms241310689)
Supplement: Supplementary file 1 [file ijms-24-10689-s001.zip › ijms-2454614-supplementary.pdf]

Supplementary material S1

Experimental treatment with edaravone in a mouse model of spinocerebellar ataxia 1
Martina Sucha, Simona Benediktova, Filip Tichanek, Jan Jedlicka, Stepan Kapl, Dana Jelinkova, Zdenka Purkartova, Jan Tuma, Jitka Kuncova, Jan Cendelin

List of gait parameters measured using CatWalk and their correlations with individual principal components

Parameter	PC1_cor	PC2_cor	PC3_cor
Run_Average_Speed_.cm.s._SD	0.27	0.61	0.36
RF_Stand_.s._SD	-0.66	0.65	0.11
RF_Swing_.s._SD	-0.50	0.53	0.44
RF_SwingSpeed_.cm.s._SD	0.23	0.89	0.19
RF_StrideLength_.cm._SD	-0.16	0.82	0.28
RH_Stand_.s._SD	-0.55	0.66	0.00
RH_Swing_.s._SD	-0.52	0.62	0.26
RH_SwingSpeed_.cm.s._SD	0.24	0.86	0.22
RH_StrideLength_.cm._SD	-0.19	0.79	0.33
LF_Stand_.s._SD	-0.63	0.65	0.09
LF_Swing_.s._SD	-0.42	0.51	0.55
LF_SwingSpeed_.cm.s._SD	0.16	0.90	0.11
LF_StrideLength_.cm._SD	-0.21	0.80	0.32
LH_Stand_.s._SD	-0.66	0.65	-0.05
LH_Swing_.s._SD	-0.33	0.61	0.45
LH_SwingSpeed_.cm.s._SD	0.25	0.84	0.01
LH_StrideLength_.cm._SD	-0.07	0.76	0.42
Run_Average_Speed_.cm.s._Mean	0.90	0.34	0.11
RF_Stand_.s._Mean	-0.91	-0.19	0.06
RF_Swing_.s._Mean	-0.52	-0.64	0.50
RF_SwingSpeed_.cm.s._Mean	0.77	0.54	-0.13
RF_StrideLength_.cm._Mean	0.77	-0.22	0.40
RH_Stand_.s._Mean	-0.79	-0.11	-0.07
RH_Swing_.s._Mean	-0.62	-0.48	0.44
RH_SwingSpeed_.cm.s._Mean	0.81	0.46	-0.11
RH_StrideLength_.cm._Mean	0.76	-0.20	0.39
LF_Stand_.s._Mean	-0.92	-0.18	0.02
LF_Swing_.s._Mean	-0.44	-0.65	0.53
LF_SwingSpeed_.cm.s._Mean	0.75	0.56	-0.21
LF_StrideLength_.cm._Mean	0.77	-0.23	0.39
LH_Stand_.s._Mean	-0.84	-0.06	-0.13

LH_Swing_.s._Mean	-0.53	-0.51	0.51
LH_SwingSpeed_.cm.s._Mean	0.78	0.48	-0.23
LH_StrideLength_.cm._Mean	0.75	-0.18	0.41
StepSequence_CA_...	0.10	-0.13	0.21
StepSequence_CB_...	0.34	-0.11	0.36
StepSequence_AA_...	0.21	-0.29	0.48
StepSequence_AB_...	-0.36	0.27	-0.54
Support_Zero_...	0.28	0.01	0.60
Support_Single_...	0.24	-0.25	0.76
Support_Diagonal_...	0.42	-0.51	-0.27
Support_Girdle_...	-0.29	0.19	-0.11
Support_Lateral_...	0.08	0.11	0.44
Support_Three_...	-0.52	0.49	-0.58
Support_Four_...	-0.49	0.54	-0.38
BOS_FrontPaws_Mean_.cm.	-0.05	0.09	-0.07
BOS_HindPaws_Mean_.cm.	-0.10	-0.50	0.19
PrintPositions_RightPaws_Mean_.cm.	-0.68	-0.22	0.17
PrintPositions_LeftPaws_Mean_.cm.	-0.65	-0.23	0.18

Supplementary material S2

Experimental treatment with edaravone in a mouse model of spinocerebellar ataxia 1

Martina Sucha, Simona Benediktova, Filip Tichanek, Jan Jedlicka, Stepan Kapl, Dana Jelinkova, Zdenka Purkartova, Jan Tuma, Jitka Kuncova, Jan Cendelin

Median values of individual gait parameters measured using CatWalk

Presented as median (quartile 1, quartile 3)

N...number of mice

p-value...statistical significance level of the effect of experimental group estimated by the Kruskal-Wallis rank sum test

q-value...p-value adjusted using False discovery rate correction for multiple testing

Experimental groups:
- WT_0...saline-treated wild type mice
- WT_E...edaravone-treated wild type mice
- SCA1_0...saline-treated SCA1 mice
- SCA1_E...edaravone-treated SCA1 mice

parameter	**WT_0**, N = 20	**WT_E**, N = 20	**SCA1_0**, N = 20	**SCA1_E**, N = 20	p-value	q-value
Run_Average_Speed_.cm.s._SD	7.4 (3.7, 10.9)	8.0 (4.6, 10.2)	4.9 (2.6, 7.4)	5.8 (4.4, 8.0)	0.4	0.5
RF_Stand_.s._SD	0.036 (0.027, 0.045)	0.042 (0.027, 0.047)	0.044 (0.034, 0.061)	0.037 (0.032, 0.052)	0.3	0.4
RF_Swing_.s._SD	0.026 (0.018, 0.033)	0.028 (0.024, 0.036)	0.028 (0.022, 0.035)	0.026 (0.021, 0.030)	0.8	0.8
RF_SwingSpeed_.cm.s._SD	16 (8, 22)	18 (12,29)	20 (16,23)	22 (18, 28)	0.13	0.2
RF_StrideLength_.cm._SD	0.76 (0.61, 1.09)	0.87 (0.65, 1.14)	0.88 (0.60, 1.37)	0.99 (0.76, 1.18)	0.7	0.8
RH_Stand_.s._SD	0.04 (0.03, 0.04)	0.04 (0.03, 0.05)	0.04 (0.03, 0.07)	0.03 (0.03, 0.06)	0.3	0.4
RH_Swing_.s._SD	0.029 (0.025, 0.042)	0.036 (0.026, 0.043)	0.042 (0.028, 0.054)	0.039 (0.025, 0.059)	0.4	0.5
RH_SwingSpeed_.cm.s._SD	13 (7, 18)	14 (10, 21)	14 (14, 18)	16 (11, 20)	0.6	0.7
RH_StrideLength_.cm._SD	0.80 (0.68, 1.19)	1.02 (0.75, 1.11)	0.94 (0.71, 1.31)	1.00 (0.75, 1.32)	0.6	0.7
LF_Stand_.s._SD	0.03 (0.02, 0.05)	0.04 (0.03, 0.05)	0.04 (0.03, 0.07)	0.04 (0.03, 0.06)	0.3	0.4

LF_Swing_.s._SD	0.025 (0.019, 0.036)	0.029 (0.022, 0.033)	0.027 (0.022, 0.034)	0.025 (0.021, 0.032)	0.9	0.9
LF_SwingSpeed_.cm.s._SD	17 (8, 23)	20 (14, 25)	21 (16, 24)	24 (16, 28)	0.2	0.3
LF_StrideLength_.cm._SD	0.73 (0.61, 1.14)	0.86 (0.73, 1.14)	0.86 (0.67, 1.27)	1.01 (0.71, 1.29)	0.6	0.7
LH_Stand_.s._SD	0.032 (0.026, 0.042)	0.039 (0.026, 0.046)	0.038 (0.032, 0.072)	0.040 (0.033, 0.063)	0.2	0.3
LH_Swing_.s._SD	0.028 (0.024, 0.046)	0.032 (0.026, 0.045)	0.038 (0.029, 0.050)	0.036 (0.028, 0.052)	0.4	0.5
LH_SwingSpeed_.cm.s._SD	13 (7, 17)	13, (10, 16)	14 (10, 18)	18 (11, 22)	0.2	0.4
LH_StrideLength_.cm._SD	0.90 (0.70, 1.19)	0.88 (0.71, 1.23)	0.96 (0.65, 1.25)	1.10 (0.72, 1.29)	0.8	0.9
Run_Average_Speed_.cm.s._Mean	26 (23, 28)	28 (22, 31)	23 (18, 27)	27 (23, 32)	0.11	0.2
RF_Stand_.s._Mean	0.15 (0.14, 0.16)	0.14 (0.13, 0.15)	0.14 (0.13, 0.15)	0.15 (0.12, 0.16)	0.048	0.12
RF_Swing_.s._Mean	0.128 (0.118, 0.138)	0.114 (0.109, 0.131)	0.112 (0.094, 0.139)	0.093 (0.082, 0.123)	0.006	0.04
RF_SwingSpeed_.cm.s._Mean	55 (51, 62)	65 (55, 71)	59 (48, 73)	70 (59, 82)	0.04	0.12
RF_StrideLength_.cm._Mean	6.67 (6.34, 7.02)	6.95 (6.31, 7.30)	6.31 (5.91, 6.62)	6.35 (6.18, 6.75)	0.017	0.074
RH_Stand_.s._Mean	0.119 (0.108, 0.126)	0.112 (0.103, 0.120)	0.129 (0.111, 0.160)	0.108 (0.086, 0.118)	0.048	0.12
RH_Swing_.s._Mean	0.16 (0.15, 0.18)	0.15 (0.13, 0.17)	0.15 (0.14, 0.19)	0.14 (0.11, 0.18)	0.4	0.5
RH_SwingSpeed_.cm.s._Mean	44 (40, 51)	51 (43, 58)	43 (34, 52)	51 (40, 62)	0.3	0.4
RH_StrideLength_.cm._Mean	6.63 (6.25, 7.01)	6.92 (6.32, 7.38)	6.31 (5.96, 6.59)	6.37 (6.14, 6.90)	0.022	0.091
LF_Stand_.s._Mean	0.15 (0.13, 0.16)	0.14 (0.13, 0.15)	0.17 (0.14, 0.19)	0.15 (0.12, 0.16)	0.066	0.14
LF_Swing_.s._Mean	0.127 (0.118, 0.141)	0.118 (0.109, 0.128)	0.106 (0.092, 0.142)	0.099 (0.084, 0.117)	0.003	0.025
LF_SwingSpeed_.cm.s._Mean	55 (51, 64)	65 (53, 70)	59 (47, 71)	70 (61, 85)	0.04	0.12
LF_StrideLength_.cm._Mean	6.65 (6.25, 7.07)	6.88 (6.38, 7.34)	6.33 (5.91, 6.58)	6.37 (6.14, 6.77)	0.012	0.06
LH_Stand_.s._Mean	0.120 (0.107, 0.138)	0.113 (0.095, 0.122)	0.125 (0.110, 0.149)	0.106 (0.094, 0.120)	0.061	0.14
LH_Swing_.s._Mean	0.15 (0.15, 0.18)	0.15 (0.14, 0.17)	0.14 (0.13, 0.19)	0.14 (0.11, 0.16)	0.3	0.4

LH_SwingSpeed_.cm.s._Mean	45 (40, 50)	48 (43, 57)	44 (34, 54)	52 (41, 62)	0.3	0.4
LH_StrideLength_.cm._Mean	6.61 (6.30, 7.04)	6.98 (6.36, 7.29)	6.25 (5.99, 6.58)	6.34 (6.10, 6.89)	0.011	0.06
StepSequence_CA_...	23 (14, 35)	23 (13, 35)	10 (8, 19)	13 (7, 19)	0.025	0.1
StepSequence_CB_...	22 (12, 28)	22 (13, 34)	21 (7, 26)	14 (8, 23)	0.4	0.5
StepSequence_AA_...	12 (5, 22)	11 (8, 14)	1 (0, 5)	1 (0, 6)	<0.001	<0.001
StepSequence_AB_...	38 (20, 57)	34 (25, 48)	63 (53, 74)	65 (54, 78)	<0.001	<0.001
Support_Zero_...	0.06 (0.00, 0.64)	0.19 (0.00, 0.49)	0.11 (0.00, 0.17)	0.00 (0.00, 0.12)	0.13	0.2
Support_Single_...	13 (9, 18)	16 (11, 18)	9 (5, 15)	8 (6, 14)	0.055	0.13
Support_Diagonal_...	74 (71, 78)	72 (69, 74)	69 (62, 73)	64 (61, 74)	0.01	0.06
Support_Girdle_...	3.20 (2.43, 5.02)	3.71 (3.07, 5.22)	5.86 (4.49, 7.14)	6.80 (4.64, 8.98)	<0.001	0.006
Support_Lateral_...	2.16 (1.47, 3.06)	2.52 (1.61, 3.53)	1.23 (0.76, 1.96)	1.70 (1.33, 2.40)	0.047	0.12
Support_Three_...	7 (5, 8)	4 (4, 6)	11 (7, 19)	12 (9, 15)	<0.001	<0.001
Support_Four_...	0.31 (0.10, 0.62)	0.07 (0.00, 0.35)	0.93 (0.18, 3.25)	0.39 (0.11, 1.53)	0.031	0.11
BOS_FrontPaws_Mean_.cm.	1.23 (1.15, 1.31)	1.17 (1.12, 1.26)	1.12 (1.07, 1.24)	1.12 (1.04, 1.16)	0.049	0.12
BOS_HindPaws_Mean_.cm.	2.22 (2.09, 2.36)	2.21 (2.12, 2.28)	2.21 (2.00, 2.46)	2.16 (2.06, 2.28)	0.7	0.8
PrintPositions_RightPaws_Mean_.cm.	0.72 (0.47, 0.95)	0.52 (0.32, 0.63)	0.58 (0.26, 0.85)	0.43 (0.26, 0.57)	0.12	0.2
PrintPositions_LeftPaws_Mean_.cm.	0.69 (0.56, 0.92)	0.49 (0.37, 0.63)	0.54 (0.40, 0.85)	0.33 (0.18, 0.48)	0.004	0.035
